# Alternative Immunomodulatory Strategies for Xenotransplantation: CD80/CD86-CTLA4 Pathway-Modified Immature Dendritic Cells Promote Xenograft Survival

**DOI:** 10.1371/journal.pone.0069640

**Published:** 2013-07-29

**Authors:** Min Tian, Yi Lv, Chao Zhai, Haitao Zhu, Liang Yu, Bo Wang

**Affiliations:** Department of Hepatobiliary Surgery, First Affiliated Hospital, Medical College, Xi'an Jiaotong University, Xi'an, People's Republic of China; Université Paris Descartes, France

## Abstract

**Background:**

Xenotransplantation is a promising approach to circumventing the current organ shortage. However, T-cell-dependent anti-xenoresponses are a major challenge to successful xenografts. Given the advantages of the use of CTLA4-Ig in the survival of allografts, the purpose of the study was to investigate the therapeutic potential of CTLA4-IgG4 modified immature dendritic cells (imDCs) in the prevention of islets xenograft rejection.

**Methods:**

CTLA4-IgG4 was constructed by the fusion of the extracellular regions of porcine CTLA4 to human the hIgG4 Fc region. The imDCs were induced and cultured from porcine peripheral blood mononuclear cells (PBMC). The CTLA4-IgG4 modified imDCs were delivered via the portal vein to the liver of diabetic mice (insulin-dependent diabetes mellitus) before islet xenografting, and mCTLA4-Ig was administered intravenously after xenotransplantation.

**Results:**

The xenograft survival of mice receiving unmodified imDCs was approximately 30 days. However, following administration of CTLA4-IgG4 modified imDCs before grafting and mCTLA4-Ig after grafting, xenografts survived for more than 100 days. Flow cytometric analysis showed that the CD4^+^CD25^+^Foxp3^+^ Treg population was increased in spleens. The efficacy of donor CTLA4-IgG4 modified imDCs correlated partially with the amplification of Tregs.

**Conclusions:**

These results confirm that selective inhibition of the direct and indirect pathways of T-cell activation by donor CTLA4-IgG4 modified imDCs and receptor CTLA4-Ig is a highly effective strategy to promote survival of xenografts.

## Introduction

Xenotransplantation is a promising approach to circumvent the current organ shortage. Specifically, pig has been considered the most suitable xenogeneic source for clinical islet transplantation [1]–[4]. Hyperacute rejection, however, is the greatest barrier to successful xenotransplantation in humans following this strategy, which substantially jeopardizes the xenograft survival. To address this, alpha 1,3-galactosyltransferase-knockout pigs were generated [5] and with the knockout pigs as the xenogeneic source, prolonged xenograft survival was observed in non-human primates [6]. Since evidence showed that the predominant epitope Gal(1–3) is expressed on pig endothelial cells with little expression on pig islets [7], this suggests that porcine islets may not undergo hyperacute rejection. However, the cell-mediated xenogeneic immune response is still a major challenge to successful xenotransplantation [4]. Koulmanda [8] reported that xenografts were rejected within 7 to 14 days of fetal porcine islet transplantation in mice. Xenograft survival was prolonged to 35 days in the class II-, CD4^+^ mice. Moreover, islet survival reached approximately100 days in CD4^+^ T-cell-depleted mice. These results suggested that cell-mediated xenogeneic immune responses plays a central role in successful xenotransplantation of porcine islets either by the direct pathway comprising donor antigen presenting cell (APC)-dependent responses or by the indirect pathway comprising host APC-dependent responses [9].

Dendritic cells (DCs) are crucial regulators of immunity with their ability to induce and maintain allogeneic tolerance. It is now well-recognized that immature, ‘alternatively activated’, genetically modified, maturation-resistant DCs of either donor or host origin, can promote allograft survival [10], [11]. However, some DC subtypes in a tolerogenic state are unstable. In steady-state gene-modified DCs, some regulatory molecules, including IL-10, TNF-α, PD-1L, CTLA-4 and heme oxygenase-1 have been identified [12]–[16]. Moreover, it has been reported that CD4^+^ memory T-cell responses can also be terminated when cognate antigen is transgenically expressed in steady-state DCs [17] or by a combination of blocking co-stimulatory molecules [18].

CTLA4-Ig has been extensively used to facilitate immune tolerance in allotransplants by inhibition of CD80/CD86-CD28 co-stimulatory interactions, which block T-cell activation. CD4^+^ CD25^+^ regulatory T cells (Tregs) have been shown to be important in the maintenance of tolerance, especially transplantation tolerance [19]. Several studies have shown that CD80/CD86-CD28 co-stimulatory signals, DCs, and TGF-β are necessary for the generation of the CD4^+^CD25^+^ Tregs [20]–[22]. Accordingly, by blocking the CD80/CD86-CD28 co-stimulatory interaction, CTLA4-Ig may inhibit CD4^+^CD25^+^ Treg generation. Nevertheless, the effects of a fusion protein comprising CTLA4 and the IgG4 Fc fragment on CD4^+^CD25^+^ Tregs are poorly understood. It was anticipated that CTLA4-IgG4 is likely to improve the long-term survival of xenografts due to the long half-life in the blood and the inactivation of the classical complement pathway by the IgG4 Fc fragment.

In this study, we hypothesize that, by pre-treating gene-modified donor imDCs with a pCTLA4-IgG4 fusion protein, recipients receiving gene-modified imDC injection will exhibit effective suppression of T-cell activation via the direct pathway with little suppression of the indirect pathway following islet xenotransplantation.

## Materials and Methods

### PCR and Constructs Preparation

Total RNA of porcine CTLA4 (pCTLA4) and hIgG4 Fc were isolated from pig and human peripheral blood mononuclear cells (PBMCs), respectively, using E.Z.N.ATM Total RNA Kit I (OMEGA, USA) according to the manufacturer’s instructions. RT-PCR was performed over 35 cycles with each cycle comprising 94°C for 1 min, 55°C for 1 min, extension at 72°C for 2 min and a further 7 min at 72°C. The pCTLA4 and the Hinge, CH2 and CH3 domains of the human IgG4 Fc fragment were amplified using primers based on the GenBank sequences (accession No. AF220248 and X70421). Primer sequences were as follows:

pCTLA4 Forward primer:5′-GATGTCGACAGCCATGGCTTGCTCTGGA-3′; Reverse primer:5′-GCAGAGATCTATTGATGGGAATAAAATAAGGCT-3′;

IgG4 Fc Forward primer:5′-GAGTCGACAGATCTGAATTCGAGTCCAAATCTTGTGACAAA-3′;

Reverse primer:5′-CGCTCGAGTCATTTACCCGGAGACAGGGA-3′.

The extracellular regions of pCTLA4 were PCR-amplified using pCTLA4 as a template. The same forward primer (pCTLA4) was used as for the full-length sequence. The reverse primer [23] was designed according to the 33 bases coding 11 amino acids immediately adjacent to the transmembrane region and the linked 15 bases coding a flexible linker of the regions. (:5′-CGGTTCGAATTCACCACCGGAGCCACCATCAGAATCTGGGCATGGTTCTGGATCAATGAC-3′). The 510 bp amplified product was fused to the hIgG4 regions with subcloning. The pCTLA4-IgG4 was further sub-cloned into the pShuttle-IRES-hrGFP-1 expressing vector (Stratagene, La Jolla, USA) (pShuttle-GFP-pCTLA4-IgG4).

Adv-pCTLA4-IgG4 was constructed using the AdEasyTM XL Adenoviral Vector System according to information recommended by the manufacturer (Stratagene, La Jolla, USA), and amplified using 293A cells (Baili, Shanghai,China ). Then titrated using Adeno-XTM Rapid Titer kit (Clontech), Virus titer was estimated at approximately 1×10^12^ pfu/mL by fluorescence quantitative PCR,and the stock were stored at −80°C.

### Cells and Cell Culture

Porcine monocyte-derived DCs were prepared as described in preliminary studies previously [24]. CD172a is expressed on many monocytic and granulocytic cells quite early during their differentiation. The co-expression of CD172a and CD1 combined with relatively high levels of both CD80/86 and MHC class II represent phenotypic characteristics of porcine Mo-DC [25]. Briefly, porcine PBMCs were isolated by density centrifugation (2000 r/min, 20 min) over Ficoll/Hypaque Lymphoprep(GBD,USA). Cells were washed with RPMI1640 containing 10% fetal bovine serum (FBS) and adjusted to 2 × 10^9^ cells/L before being added to 6-well plates (3 mL/well) and incubated at 37°C with 5% CO_2_ for 3 h. Non-adherent cells (more than 97% of T cells) were discarded leaving the adherent monocytes, which were maintained in fetal calf serum (FCS) supplemented with recombinant porcine IL-4 (20 ng/mL) and recombinant porcine GM-CSF (30 ng/mL) (R&D Systems, Minneapolis, USA). Half of the culture medium was replaced with the fresh medium every 48 h. Phenotypic analysis of non-adherent cells was performed by flow cytometric analysis (FACS Calibur flow cytometer) using the following antibodies: monoclonal anti-CD172a (74-22-15;Vmrd, WA, USA), monoclonal (1053h2-18-1; BD Biosciences, USA) against SLA-DR, monoclonal antibodies Foxp3 Staining Set (BD Biosciences, USA) against CD4, CD25 and Foxp3, monoclonal antibody FITC-human CD152-Ig (Ancell Corporation, Bayport, USA) ) against CD80/CD86, and FITC-goat anti-mouse IgG (Beijing Biosynthesis Biotechnology, China).

### pCTLA4-IgG4 Gene-modified Porcine imDCs

The porcine CTLA4-IgG4 adenoviral expression vector (Adv-pCTLA4-IgG4) and the control adenoviral vector (Adv-GFP) were used to transduce porcine imDCs at a multiplicity of infection (MOI) of 500∶1 at 37°C with 5% CO_2_ incubation for 24 h. Cells were then gently washed and maintained in RPMI1640 medium supplemented with 10% FBS. Samples of imDCs were identified based on the expression of pCTLA4-IgG4 and Indoleamine 2, 3-dioxygenase (IDO) by RT-PCR and Western blotting.

Specific primer pairs were as follows:

pCTLA4-IgG4: Forward: 5′-CTCCTGTACCCACCACCCTA-3′, Reverse: 5′-TGGGCATGTGTGAGTTTTGT-3′;

IDO: Forward: 5′-TAGCAAGGAGAGTGCGGATT-3′.

Reverse: 5′-TCCCTTCCAGATGATTCCAG-3′;

β-actin: Forward: 5′-GTGCGGGACATCAAGGAGAAG-3′,

Reverse: 5′-AGGAAGGAGGGCTGGAAGAG-3′.

Phenotypic analysis of pCTLA4-IgG4 modified imDCs was performed by flow cytometric analysis (FACSCalibur flow cytometer) using the following antibodies: monoclonal antibody (FITC-human CD152-Ig, 501-040) against CD80/CD86 (Ancell Corporation).

### Mixed Lymphocyte Reaction (MLR)

Mouse splenocytes were harvested to isolate mononuclear cells as responders. Porcine monocyte-derived imDCs were divided into four groups: unmodified imDCs, pCTLA4-IgG4 modified imDCs for 24 h, pCTLA4-IgG4 modified imDCs for 48 h and pCTLA4-IgG4 modified imDCs for 72 h. Responder cells (100 µl at 1×10^7^/mL) and mitomycin C pretreated stimulator cells (100 µl at 1×10^6^/mL) were seeded into 96-well plates followed by incubation at 37°C for 72 h. Simultaneously, L-tryptophan (250 µmol/L), an inhibitor of IDO, was added to another four groups of stimulator cells and responder cells. Cell proliferation was assessed by measurement of absorbance at 490 nm (A_490_) using a microtiter plate reader following the addition of MTS tetrazolium compound (Promega Corporation, USA) for 4 h. The stimulation index (SI) = (A_490_ samples - A_490_ medium control)/(A_490_ negative control - A_490_ medium control).

### Islet Xenotransplantation

#### The insulin-dependent diabetes mellitus (IDDM) model mice

Male BALB/c mice (Medical College, Xi'an Jiaotong University, China), weighing 20–25 g, were rendered diabetic by intraperitoneal injection of streptozotocin (STZ) (Sigma, USA) at a dose of 150 mg/kg. Hyperglycemia was defined as a fasting glucose level greater than 16.7 mmol/L on two consecutive occasions after STZ injection. All experiments were approved by the Medical Institutional Animal Care and Use Committee.

#### Adult porcine pancreas procurement, digestion and islet purification

Pancreases were obtained from 12 adult large white pigs (aged 12 months) with an average weight of 150 kg (Medical College, Xi'an Jiaotong University, China). Pancreatic islets were isolated and purified as described previously [26]–[29].

#### Islet xenotransplantation

The IDDM model mice served as recipients. Maestri et al. suggested that portal vein transfusion of donor-specific DCs is the optimal route [30]. After anesthesia with 10% chloric aldehyde, the mice were placed in the supine position and the abdominal was surgically opened. The imDC (1×10^7^ in 0.5 mL) or mCTLA4-Ig suspension (10 µg) was infused into the liver of mice via the portal vein using a 23-gauge needle. The following day, porcine islets (600–800 IEQ) were transplanted into the renal subcapsular space of the unilateral kidneys of the mice [29], [31]. The experimental mice were then randomly divided into seven groups (n = 15 per group).

In vivo experimental protocol was as follows:

1 Islet xenotransplantation performed with inhibition of the direct pathway of T-cell activation.

Islet xenotransplantion only (Group I, n = 15); Islet xenotransplant with injection of pCTLA4-IgG4 modified imDCs administered via the portal vein one day prior to grafting (Group II, n = 15); Islet xenotransplant with injection of hIgG4 modified imDCs administered via the portal vein one day prior to grafting (Group III, n = 15); Islet xenotransplant with injection of unmodified imDCs administered via the portal vein one day prior to grafting (Group IV, n = 15).

2 Islet xenotransplantation performed with selective inhibition of the direct and indirect pathways of T-cell activation.

Islet xenotransplant with injection of pCTLA4-IgG4 modified imDCs (group V, n = 15), mCTLA4-Ig (group IV, n = 15) administered via the portal vein one day prior to grafting, followed by intravenous administration of mCTLA4-Ig via the tail vein 7 and 14 days after grafting. As controls, islet xenotransplantation was performed with administration of pCTLA4-IgG4 modified imDCs through the portal vein one day prior to grafting, followed by intravenous administration of Adv-IgG4 (group VII, n = 15) via the tail vein 7 and 14 days after grafting.

Six healthy non-transplanted mice were used as controls. No immunosuppressive agents or insulin was administered after transplantation.

#### Xenograft survival

Non-fasting blood glucose levels and body weight were measured daily in the first week following transplantation. Subsequently, the measurements were performed three times per week. Xenograft failure was determined when the non-fasting blood glucose level exceeded 11.1 mmol/L for two consecutive days. No statistical differences in blood glucose levels or body weight were observed among the six groups before transplantation. Six mice in each group were selected at random to investigate xenograft survival times.

#### Flow cytometric analysis of CD4^+^ T cells and MLRs

Splenocytes were harvested from recipient mice at 10 days after transplantation. Mononuclear cells were isolated using the Lymphoprep method. The recipient mice comprised the islet only xenotransplantation group (Group I), the pCTLA4-IgG4 modified imDCs treatment group (Group II) and the unmodified imDCs treatment group (Group IV). CD4^+^ T cells were purified by using a CD4^+^ T-cell Isolation Kit II (Miltenyi Biotec, Germany) following the manufacturer’s instructions. CD4^+^CD25^−^ T cells and CD4^+^CD25^+^ T cells were separated using CD25 Microbeads (Miltenyi Biotec). The purity of CD4^+^CD25^+^ T cells was checked by flow cytometry. The CD4^+^ CD25^+^ T-cell suspensions were stained with PE-labeled anti-CD4, APC-labeled anti-CD25 and FITC-labeled anti-Foxp3 (eBioscience, USA), and analyzed on a FACSCalibur (BD Biosciences, USA) using CellQuest software (BD Biosciences, USA).

#### Immunohistochemical staining of the insulin and pCTLA4-IgG4

Kidney and liver samples obtained from the mice were fixed in 4% paraformaldehyde, decalcified in EDTA bone decalcifier, embedded in paraffin and sectioned at 4 µm intervals. Sections were dewaxed using xylene, dehydrated in a gradient of alcohols, and then stained using hematoxylin and eosin (H&E). Endogenous peroxidase activity was quenched with methanol in 3% H_2_O_2_. Immunohistochemistry was performed using the HistostainTM-Plus ABC kit (ZYNED, USA). Tissues were incubated with primary antibodies against insulin (ab9569) (Abcam Ltd. Hong Kong, China) and CTLA4 (52Ex) (Santa Cruz, CA, USA) overnight at 4°C. The primary antibodies were then detected with a biotinylated secondary antibody. The final colored product was developed using the DAB chromogen.

#### Western blotting

Livers and kidney samples harvested from mice were prepared in RIPA buffer (Runde, Xi’an, China) in the presence of protease inhibitors (Thermo Scientific, USA). Heat-denatured and β-mercaptoethanol-reduced samples were fractionated on 12% BisTris-SDS gradient gels (Invitrogen, USA) followed by protein transfer to nitrocellulose membrane. Then the membranes were blocked with 5% non-fat milk in 0.1% Tween 20 in Tris-buffered saline (TTBS) for 1 h and incubated with the monoclonal antibody against CTLA-4 (52Ex) (sc-73870, Santa Cruz, USA) at 1∶200 dilution for 12 h at 4°C. The membrane was washed with TTBS and incubated in goat anti-mouse IgG (H+L) FITC (Runde) for 1 h at 37°C. Detection was performed by a chemiluminescence method using the Duo/Lux kit (Vector Laboratories Inc.).

### Statistical Analysis

Statistical analysis was performed by PASW Statistics 18.0. Data are shown as mean±standard deviations. Each group was compared by One-Way ANOVA or Kaplan-Meier log-rank test. *P*-values less than 0.05 are considered to be significant.

## Results

### Morphology and Surface Molecule Profile of Porcine Monocyte-derived DCs (Mo-DCs)

To investigate the roles of CTLA4-IgG4 in cell-mediated xenogeneic immune responses, we initially prepared porcine Mo-DC as previously described [25]. During Mo-DC culture, cells were treated with rpGM-CSF plus rpIL-4 and cell morphology was observed over time. After two days of incubation, the cells appear irregular. Suspension cell proliferation was observed with cluster-like growth. Cells exhibited increased dendritic morphology with time. Suspension cells were harvested at days 5 and 9 for examination by transmission electron microscopy (Figure S1). At day 5, more than 95% of Mo-DCs at day 5 exhibited circular morphology containing intracellular vesicles with surface roughness around tiny pseudopods, while at day 9 cells exhibited typically elongated dendritic cell morphology, with asymmetric positioning of the nucleus.

Flow cytometric analysis of several surface markers was conducted to further distinguish the different Mo-DCs at days 5 and 9 (Figure S2). The results showed that, in comparison to the cells at day 5, at day 9 more cells expressed surface markers including, SLA-DR (52.32% vs. 68.09%), CD172a (54.67% vs. 82.27%) and CD80/CD86 (15.67% vs. 88.89%). These data suggested that the day 5 population contained more immature DCs (imDCs) than that at day 9. Therefore, day 5 cells (imDCs) were used in subsequent experiments.

### pCTLA4-IgG4 Modified imDCs and MLR

The Adv-pCTLA4-IgG4 (MOI: 0, 50, 100, 200, 500, 1,000) was transfected into the porcine imDCs. GFP expression was investigated by fluorescence microscopy at 48 h post-infection. The efficiencies of porcine imDCs transduction with Adv-pCTLA4-Ig was approximately 60% at MOI 200 and 90% at MOI 500. However, at MOI 1,000, the original morphology of the cells disappeared and cell-death was observed in the majority of cells. These data indicated that a MOI of 500 was optimal.

Flow cytometry analysis showed that transfected imDCs still maintained an immature phenotype as evidenced by the low levels of cell surface markers, CD80/CD86 (14.52%) (Figure S3). pCTLA4-IgG4 and IDO expression in the transfected imDCs were detected by RT-PCR and Western Bolt (Figure S4).

However, overexpression of pCTLA4-IgG4 significantly decreased the SI analysis (Figure S5, *P*<0.01). Among the cell groups, the lowest SI was observed at 48 h post-transfection, suggesting that pCTLA4-IgG4 mediated the greatest inhibition at this time-point. In contrast, addition of the IDO inhibitor, L-tryptophan, reversed the inhibition mediated by pCTLA4-IgG4, indicating that the overexpression of pCTLA4-IgG4 regulates IDO expression, which subsequently inhibits imDC proliferation.

### CD4^+^CD25^+^Foxp3^+^ Tregs in Recipient Mice

CD4^+^ T cells were purified from splenocytes of recipient mice at day 10 after transplantation. Phenotypes of these cells were characterized by detecting CD4, CD25 and Foxp3 expression (data not show). CD4 negative selection, CD4^+^CD25^+^ regulatory T cells constituted 14.62% of the total CD4^+^ cell count. CD25 positive selection, CD4^+^ T cells constituted more than 95% of the total population, while the CD4^+^CD25^+^ regulatory T cells accounted for 53.34% of the total population. The ratio of Foxp3^high^ cells in the CD4^+^CD25^+^T-cell fraction in pCTLA4-IgG4 modified imDC treated recipients (Group II) was higher than that in unmodified imDC treated recipients (Group IV) or only islet xenotransplant recipient mice (Group I) (Figure S6, *P*<0.01).These findings suggest that the pCTLA4-IgG4 modified imDCs promote CD4^+^CD25^+^Foxp3^+^ Treg cell differentiation and/or expansion.

### Histochemical Staining of Xenografts

In each of Groups I to VI (see Materials and Methods), xenografts were examined by H&E histochemical staining. Intact liver structures were observed in Group I (Figure S7A). In Groups II and V, positive pCTLA4-IgG4 expression was clearly detected (Figure S7B, Figure S7E), while no expression was observed in the other groups (Figure S7C, Figure S7D, Figure S7F, Figure S7G). Western blotting analysis of pCTLA4-IgG4 protein expression (43 kD) in different tissues in the transplanted mice revealed positive expression in the liver and kidney tissues in Groups II and V but not in Group VI (Figure S7H). These findings suggest that overexpression of the pCTLA4-IgG4 fusion protein is a feasible strategy to improve the success of xenograft survival.

### Xenograft Surviva

Analysis revealed that xenograft survival time was significantly prolonged in the pCTLA4-IgG4 modified imDCs injection groups (Groups II) compared to the other groups (Group I, Group III and Group IV) (Figure S8, *P*<0.01). These findings demonstrated that the pCTLA4-IgG4 modified imDCs efficiently blocked the direct pathway of T-cell activation.

In contrast, xenografts were rejected by day 20 in all mice treated with mCTLA4-Ig before and after grafting. Furthermore, no obvious differences in xenograft survival were observed between the mice injected with pCTLA4-IgG4 modified imDCs combined with Adv-IgG4 and those injected with only pCTLA4-IgG4 modified imDCs. However, the survival time for mice injected with pCTLA4-IgG4 modified imDCs before grafting combined with mCTLA4-Ig after grafting, was more than 100 days (Figure S9, *P*<0.01). These data suggest that pCTLA4-IgG4 modified donor imDCs combined with mCTLA4-Ig may function to block the direct and indirect pathways of T-cell activation.

## Discussion

With CD4^+^ cells as the main effector cells, the major obstacle to successful islet xenotransplantation is the T-cell mediated immune response. For example, porcine skin xenografts in splenectomized mice were always rejected in a T-cell-dependent fashion [32]. CD4^+^ T-cell-mediated xenotransplantation resistance involves two distinct pathways: the direct pathway involving donor antigen presenting cell (APC)-dependent responses and the indirect pathway involving host APC-dependent responses. It has been shown in vitro that the direct pathway plays an important role in the initiation of primary T-cell responses to xenografts [33], while several in vivo studies have demonstrated that the indirect pathway also plays a role in xenograft rejection [34]–[37]. However, the precise mechanisms of immune regulation of xenograft transplantation in vivo remain unclear.

Exogenous CTLA4-Ig gene transfer has been demonstrated to induce immune tolerance in vivo [38]–[39], with multiple factors contributing to the overall function of CTLA4 in transplant immune modulation [40]. First, it was demonstrated that CTLA-4 reduces the contact between T cells and APCs and leads to a decrease in pro-inflammatory cytokine production and proliferation [41]. Second, CTLA-4 prevents T-cell activation by competitive binding with CD80/CD86 molecules on the APCs with a binding affinity 100 times greater than that of CD28 for CD80/CD86 [42]. Although Vaughan [23] showed that both pCTLA4-Ig and hCTLA4-Ig is capable of blocking human CD4^+^ T-cell proliferation, the binding affinity of pCTLA4-Ig to human CD80/CD86 is low and pCTLA4-Ig fails to inhibit human CD4^+^ T-cell responses co-stimulated by human B7, suggesting that pCTLA4-Ig may be effective in inhibiting the direct pathway in xenotransplantation. Lastly, CTLA4 induces DCs to express indoleamine-2,3-dioxygenase (IDO), which catalyzes the degradation of tryptophan, an essential stimulus for effector T-cell apoptosis in a tryptophan-deprived environment [43], [44].

In pilot studies of the overexpression of pCTLA4-IgG4 (Fc) fusion protein, MLRs revealed that the inhibition of proliferation of xenogeneic splenocytes was more efficient in the pCTLA4-IgG4 gene-modified imDCs group than that in unmodified imDCs group [24]. Others have shown that the survival of donor-derived (porcine) CTLA4-IgG4 gene-modified porcine islet xenografts was significantly prolonged in rats with diabetes. Additionally, we found that the level of IFN-γ decreased and IL-4 increased in the pCTLA4-IgG4 gene-modified porcine islet xenograft group [29]. It can be speculated that the pCTLA-IgG4 fusion protein blocks the direct pathway of recipient T-cell priming, which eventually induces the differentiation bias of T helper (Th) cells, which might be responsible for the significant prolongation of xenograft survival. Taken together, these results suggest that overexpression of the pCTLA4-IgG4 fusion protein is a feasible strategy to improve the success of xenograft survival.

In this study, we showed that islet xenograft survival in the control group without pCTLA4-IgG4 overexpression was only about 7 days. In contrast, xenograft survival following pre-infusion of donor unmodified imDCs was prolonged to more than 30 days. Xenograft survival following pre-infusion of donor pCTLA4-IgG4 modified imDCs was significantly prolonged to more than 60 days. Furthermore, the xenograft survival in mice treated with a pre-infusion of pCTLA4-IgG4 modified imDCs before grafting and mCTLA4-Ig injected late after grafting was more than 100 days. Furthermore, flow cytometric analysis showed that the CD4^+^CD25^+^Foxp3^+^ Treg population in the spleen was increased in the pCTLA4-IgG4 modified imDCs group. It is possible that pCTLA4-IgG4 modified imDCs expressed pCTLA4-Ig which may be effective in inhibiting direct the pathway of CD4^+^T-cell activation in islet xenotransplantation. Another possibility may be that the pCTLA4-IgG4 modified imDCs, known as tolerogenic DCs, which expressed IDO, subsequently induced effector T-cell apoptosis [44]. Moreover, another potentially important aspect is that CD4^+^CD25^+^Foxp3^+^Treg cell differentiation and/or expansion is promoted in the recipient pretreated with pCTLA4-IgG4 modified imDCs. Many studies have confirmed the ability of CD4^+^ CD25^+^ Treg cells to prevent the rejection of xenogeneic grafts in vitro [45], [46]. Activated Treg cells may also down-regulate CD80/CD86 expression on host APCs, leading to the inhibition of cytokine production by DCs or IDO expression [47]–[49]. The current study implies that synergistic cross-linked interplay of pCTLA4-IgG4-modified-imDC and Tregs may play a critical role in transplantation tolerance. Recently, heme oxygenase-1 (HO-1) was identified as a marker of (T-cell mediated) injury as well as an indicator of beneficial effects [50]. Therefore, further investigations will be required to determine the efficacy of tolerance induction using donor-specific transfusions and co-stimulation blockade in xenotransplantation, especially since there could be a difference in efficacy among low and high HO-1 expressing recipients.

In summary, our studies have demonstrated that pCTLA4-IgG4 modified donor imDCs significantly prolong the survival of islet xenografts. It is hypothesized that the underlying mechanisms of this effect involve efficient blockade of the direct pathway by direct binding of pCTLA4-IgG4 to porcine CD80/CD86 molecules on donor APCs, and induction of CD4^+^CD25^+^Foxp3^+^Treg cell proliferation. The local regulatory mechanisms of tolerogenic DC-Treg interactions may lead to anti-xenograft T-cell responses. Furthermore, the indirect pathway of CD4^+^ T-cell activation is also critical in xenograft rejection. After taking into account preferential binding of the porcine and murine CTLA4-Ig to species-matched B7 molecules [9], pCTLA4-IgG4 gene-modified donor imDCs combined with murine CTLA4-Ig blocked both the direct and indirect pathways and led to long-term xenograft survival. These results confirm that independent and selective inhibition of direct and indirect T-cell responses to porcine islet xenografts is a highly effective strategy for improving xenograft survival.

## Supporting Information

Figure S1 **Mo-DCs at day 5 and day 9 were examined by transmission electron microscopy.** A: d5 (magnification, ×6000); B d9 (magnification, ×6000).
(TIF)

Figure S2 **Expression of surface molecules on DCs at day 5 and day 9 (M1: percentage of positive cells).** A: 52.32% of these cells expressed SLA-DR; B: 54.67% of these cells expressed the myeloid differentiation antigen CD172a (SWC3); C: 15.67% of these cells expressed CD80/CD86; D: 68.09% of these cells expressed SLA-DR; E: 82.27% of these cells expressed CD172a (SWC3); F: 88.89% of these cells expressed CD80/CD86.
(TIF)

Figure S3 **Surface molecule expression on transfected imDCs at day 5.** 14.52% of these cells expressed CD80/CD86.
(TIF)

Figure S4 **RT-PCR and Western Blot identification of pCTLA4-IgG4 modified and Adv-pCTLA4-IgG4 modified imDCs.** A: Lane 1: Adv-pCTLA4-IgG4 modified imDC group, pCTLA4-IgG4 fusion gene specific fragment (approximately 144 bp); Lane 2: unmodified imDC group; Lane 3: blank control group; M: Wide Range DNA Marker (100–6,000 ); B: Lane 1: Adv-pCTLA4-IgG4 modified imDC group; Lane 2: IDO specific fragment (approximately 732 bp); M: DL15,000 Plus DNA Ladder. C: Western blot detection of pCTLA4-IgG4 expression of Adv-pCTLA4-IgG4 modified imDC. 1: control group; 2: unmodified imDC group; 3: Adv-pCTLA4-IgG4 modified imDC group; β- actin: 42 kDa.
(TIF)

Figure S5 **Stimulation index of mixed lymphocyte reaction in vitro.** *P<0.01: The stimulation indexes of pCTLA4-IgG4 modified imDCs (24 h, 48 h and 72 h) groups were significantly lower than those of unmodified imDCs group; **P<0.01: The stimulation indexes of pCTLA4-IgG4 modified imDCs (24 h, 48 h and 72 h) following the addition of L-tryptophan were higher than those without L-tryptophan.
(TIF)

Figure S6 **The Splenic CD4^+^CD25^+^Foxp3^+^ Tregs of recipient mice were Flow cytometric analyzed at day 10 after transplantation.** A: Lymphocytes lap door; B: CD4^+^CD25^+^T cells analyzed before CD4^+^ T-cell purification; C: CD4^+^CD25^+^T cells were analyzed after CD4+ T cells purification; D: Foxp3 analyzed in the CD4^+^CD25^+^ T-cell fraction; E: The population of CD4^+^CD25^+^Foxp3^+^ T cells in pCTLA4-IgG4 modified imDC recipient mice (Group II, n = 3, 26.36±1.97%) was larger than those in islet xenograft recipient mice (Group I, n = 3, 7.03±0.22%)and unmodified imDC recipient mice(Group IV, n = 3, 14.02±2.98%)(*P<0.01); FITC, fluorescein isothiocyanate; APC, allophycocyanin; PE, phycoerythrin.
(TIF)

Figure S7 **Contrast the histological of the liver and kidney between different groups at day 5 after xenotransplantation.** A: Hematoxylin and eosin (H&E) stained liver (magnification, ×100); B, E: Expression of CTLA4-IgG4 detected in Groups II and V by immunohistochemistry (arrow) (magnification, ×100); C, D, F, G: No expression of CTLA4-IgG4 was detected by immunohistochemistry in Groups III, IV and VI (magnification, ×100); H: Expression of pCTLA4-IgG4 protein in liver and kidney tissue of recipient mice detected by Western blot analysis (n = 2). Western blot analysis showing positive expression of pCTLA4-IgG4 in liver and kidney tissue of Groups II and V, and negative expression in Group VI.
(TIF)

Figure S8 **The islet xenograft survival of four groups (Group I, Group II, Group III and Group IV) (n = 6) in first experiment.** Xenograft survival in the pCTLA4-IgG4 modified imDC treated group (61.00±4.20 days, *P<0.01) was significantly longer than that in the islet only xenograft group (7.83±1.47 days), IgG4 modified imDC treated group (31.33±2.07 days), and unmodified imDC treated group (32.50±5.24 days).
(TIF)

Figure S9 **The islet xenograft survival of three groups (Group V, Group VI, Group VII) (n = 6) in the second experiment.** Xenograft survival in the pCTLA4-IgG4 modified imDC combined with mCTLA4-Ig treated group (111.83±2.71 days, **P<0.01) was significantly longer than that in the mCTLA4-Ig combined with mCTLA4-Ig treated group (21.67±1.75 days), and the pCTLA4-IgG4 modified imDC combined with Adv-IgG4 treated group (60.17±1.94 days).
(TIF)
