# Supplementary figures and images for: Alternative Immunomodulatory Strategies for Xenotransplantation: CD80/CD86-CTLA4 Pathway-Modified Immature Dendritic Cells Promote Xenograft Survival
Source: PLoS One. 2013 Jul 29;8(7):e69640. doi: 10.1371/journal.pone.0069640 (PMC3726660; doi:10.1371/journal.pone.0069640)

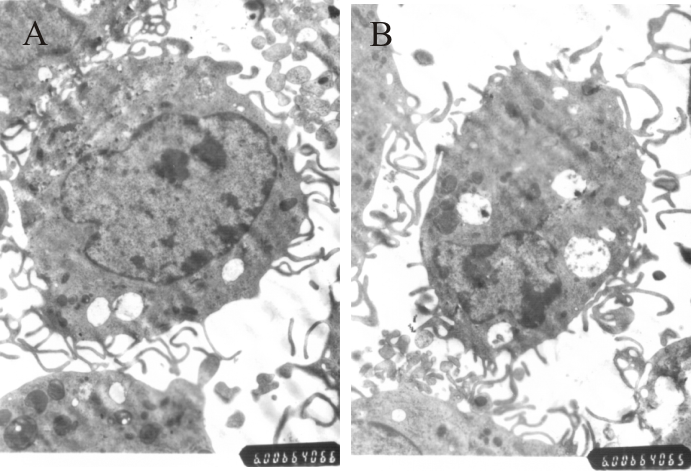

Supplement: Figure S1 — Mo-DCs at day 5 and day 9 were examined by transmission electron microscopy. A: d5 (magnification, ×6000); B d9 (magnification, ×6000). (TIF) [file pone.0069640.s001.tif]

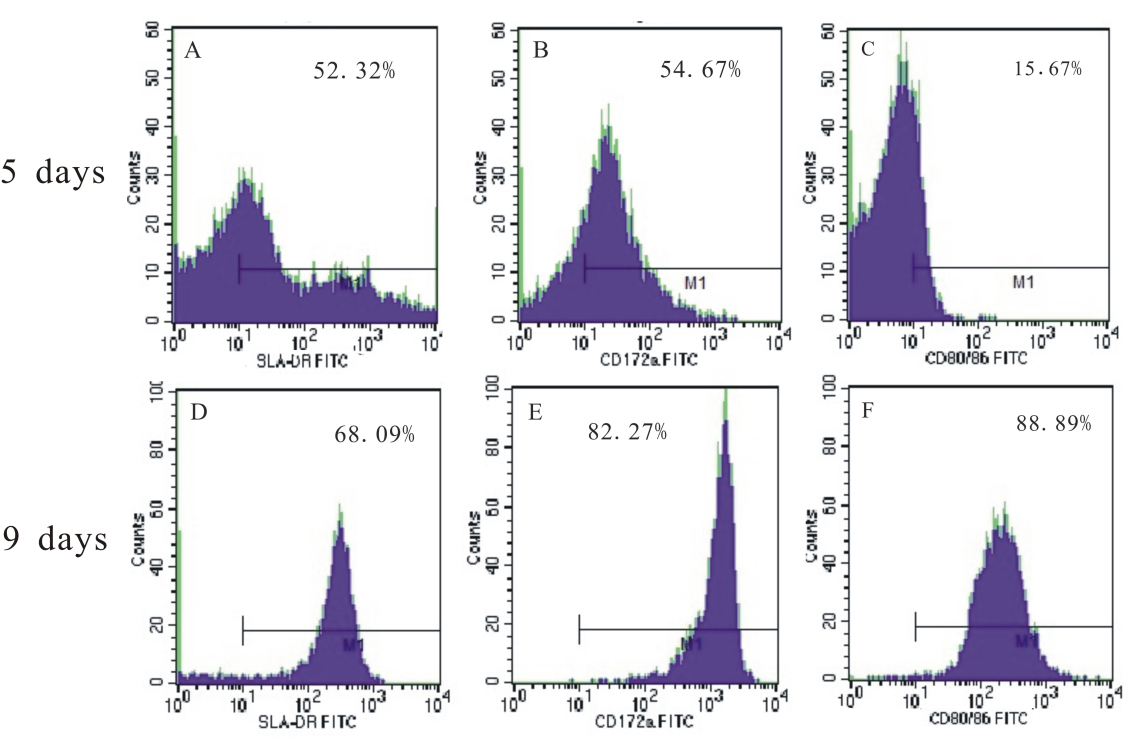

Supplement: Figure S2 — Expression of surface molecules on DCs at day 5 and day 9 (M1: percentage of positive cells). A: 52.32% of these cells expressed SLA-DR; B: 54.67% of these cells expressed the myeloid differentiation antigen CD172a (SWC3); C: 15.67% of these cells expressed CD80/CD86; D: 68.09% of these cells expressed SLA-DR; E: 82.27% of these cells expressed CD172a (SWC3); F: 88.89% of these cells expressed CD80/CD86. (TIF) [file pone.0069640.s002.tif]

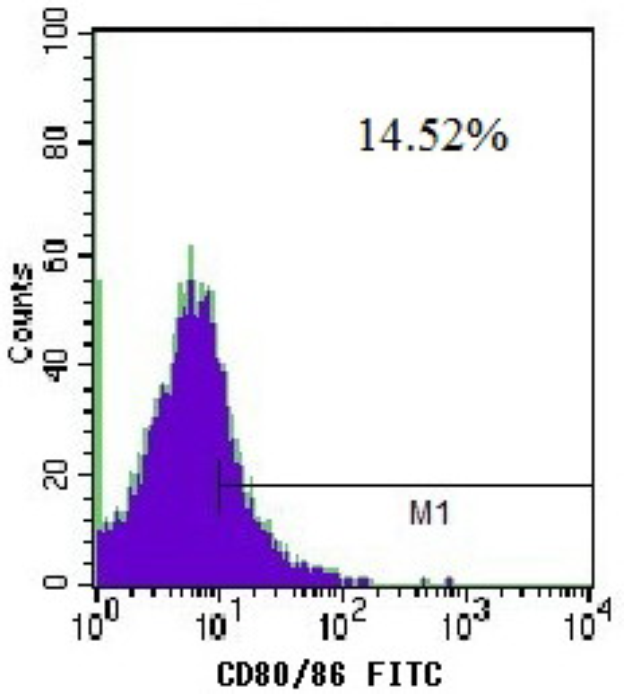

Supplement: Figure S3 — Surface molecule expression on transfected imDCs at day 5. 14.52% of these cells expressed CD80/CD86. (TIF) [file pone.0069640.s003.tif]

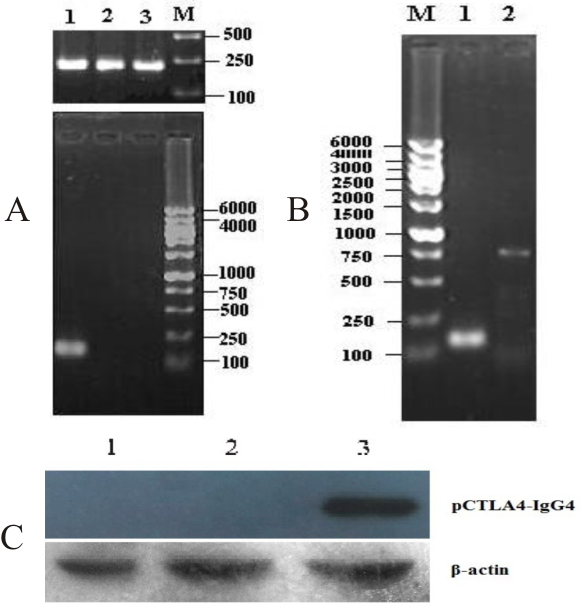

Supplement: Figure S4 — RT-PCR and Western Blot identification of pCTLA4-IgG4 modified and Adv-pCTLA4-IgG4 modified imDCs. A: Lane 1: Adv-pCTLA4-IgG4 modified imDC group, pCTLA4-IgG4 fusion gene specific fragment (approximately 144 bp); Lane 2: unmodified imDC group; Lane 3: blank control group; M: Wide Range DNA Marker (100–6,000 ); B: Lane 1: Adv-pCTLA4-IgG4 modified imDC group; Lane 2: IDO specific fragment (approximately 732 bp); M: DL15,000 Plus DNA Ladder. C: Western blot detection of pCTLA4-IgG4 expression of Adv-pCTLA4-IgG4 modified imDC. 1: control group; 2: unmodified imDC group; 3: Adv-pCTLA4-IgG4 modified imDC group; β- actin: 42 kDa. (TIF) [file pone.0069640.s004.tif]

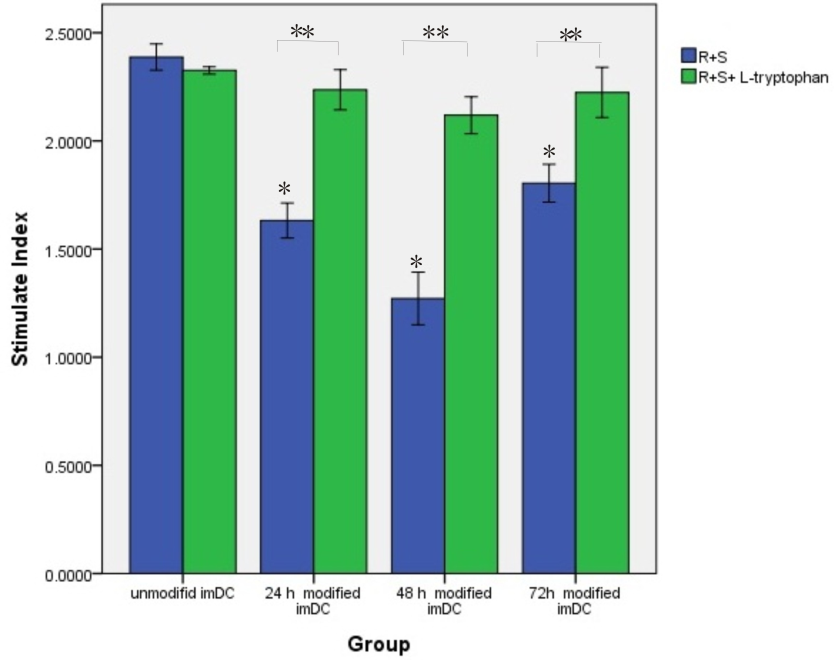

Supplement: Figure S5 — Stimulation index of mixed lymphocyte reaction in vitro. *P<0.01: The stimulation indexes of pCTLA4-IgG4 modified imDCs (24 h, 48 h and 72 h) groups were significantly lower than those of unmodified imDCs group; **P<0.01: The stimulation indexes of pCTLA4-IgG4 modified imDCs (24 h, 48 h and 72 h) following the addition of L-tryptophan were higher than those without L-tryptophan. (TIF) [file pone.0069640.s005.tif]

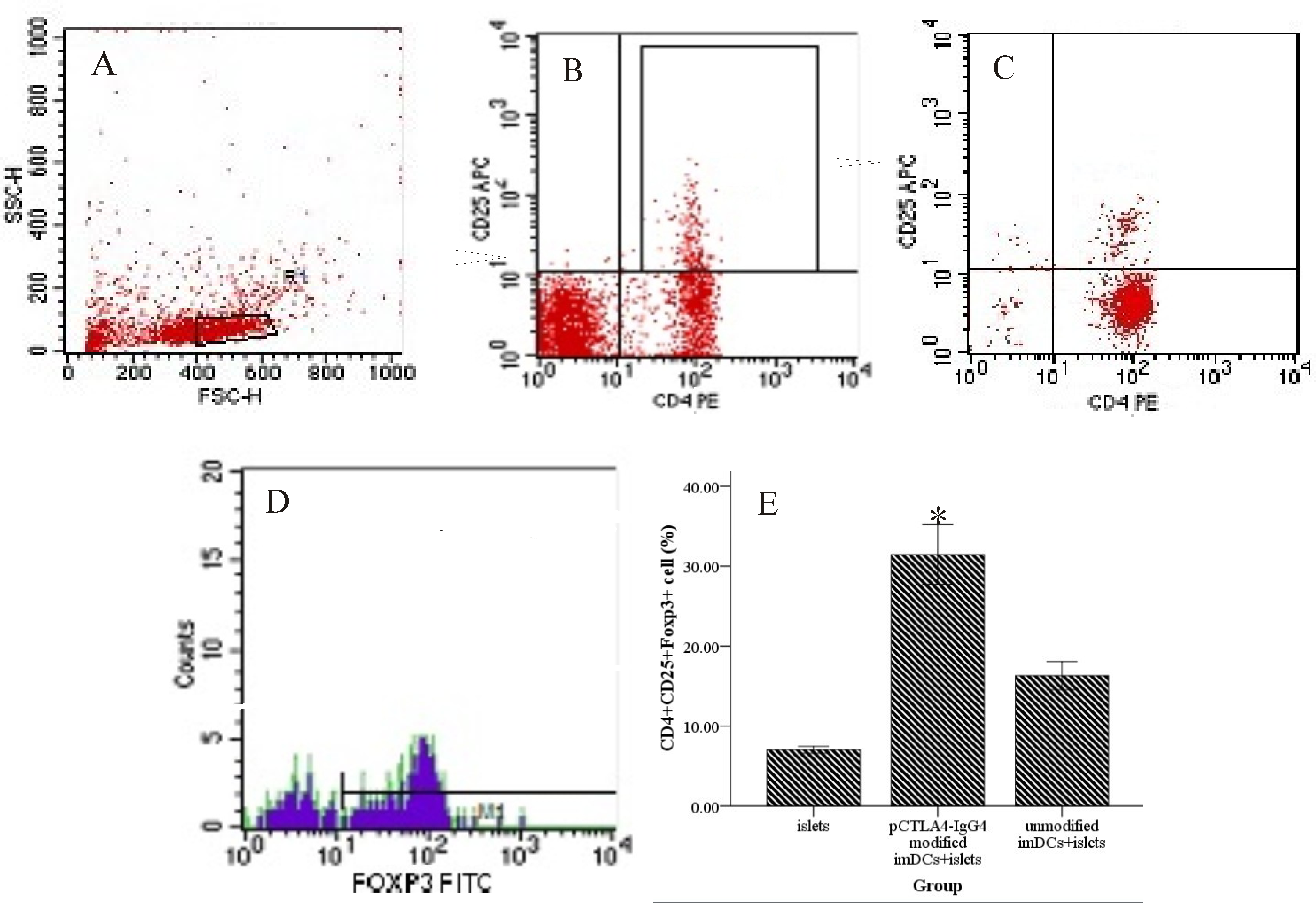

Supplement: Figure S6 — The Splenic CD4+CD25+Foxp3+ Tregs of recipient mice were Flow cytometric analyzed at day 10 after transplantation. A: Lymphocytes lap door; B: CD4+CD25+T cells analyzed before CD4+ T-cell purification; C: CD4+CD25+T cells were analyzed after CD4+ T cells purification; D: Foxp3 analyzed in the CD4+CD25+ T-cell fraction; E: The population of CD4+CD25+Foxp3+ T cells in pCTLA4-IgG4 modified imDC recipient mice (Group II, n = 3, 26.36±1.97%) was larger than those in islet xenograft recipient mice (Group I, n = 3, 7.03±0.22%)and unmodified imDC recipient mice(Group IV, n = 3, 14.02±2.98%)(*P<0.01); FITC, fluorescein isothiocyanate; APC, allophycocyanin; PE, phycoerythrin. (TIF) [file pone.0069640.s006.tif]

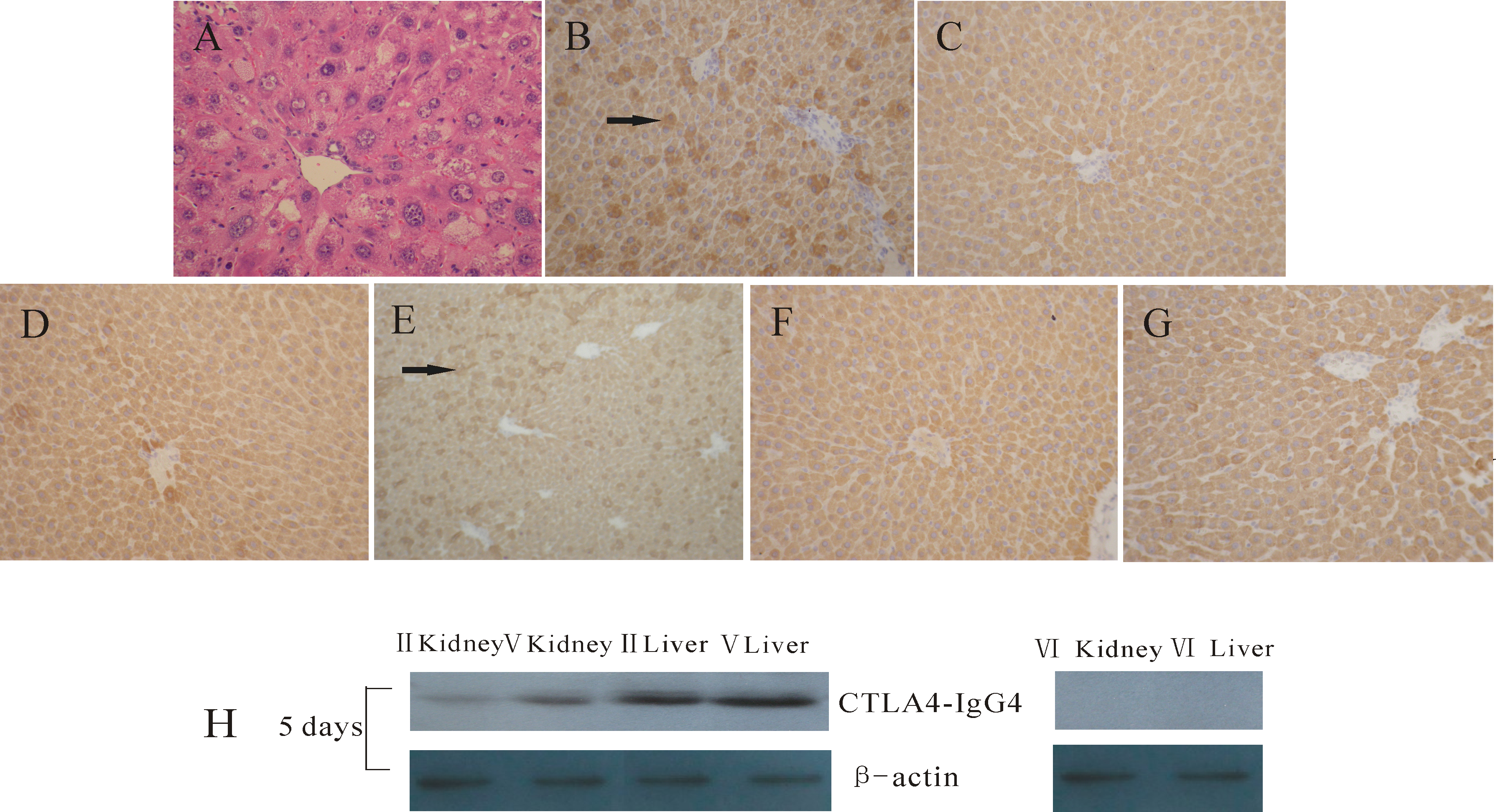

Supplement: Figure S7 — Contrast the histological of the liver and kidney between different groups at day 5 after xenotransplantation. A: Hematoxylin and eosin (H&E) stained liver (magnification, ×100); B, E: Expression of CTLA4-IgG4 detected in Groups II and V by immunohistochemistry (arrow) (magnification, ×100); C, D, F, G: No expression of CTLA4-IgG4 was detected by immunohistochemistry in Groups III, IV and VI (magnification, ×100); H: Expression of pCTLA4-IgG4 protein in liver and kidney tissue of recipient mice detected by Western blot analysis (n = 2). Western blot analysis showing positive expression of pCTLA4-IgG4 in liver and kidney tissue of Groups II and V, and negative expression in Group VI. (TIF) [file pone.0069640.s007.tif]

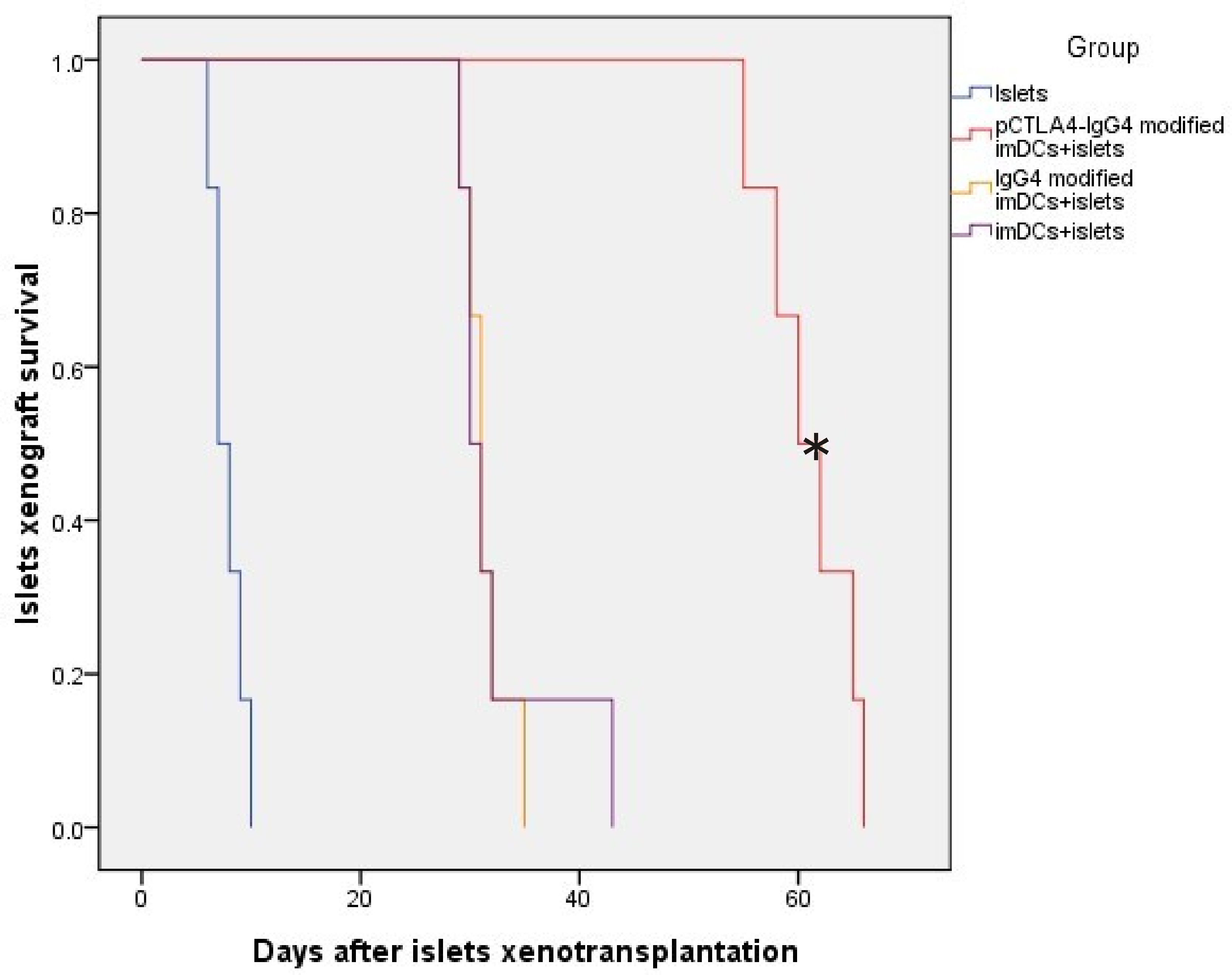

Supplement: Figure S8 — The islet xenograft survival of four groups (Group I, Group II, Group III and Group IV) (n = 6) in first experiment. Xenograft survival in the pCTLA4-IgG4 modified imDC treated group (61.00±4.20 days, *P<0.01) was significantly longer than that in the islet only xenograft group (7.83±1.47 days), IgG4 modified imDC treated group (31.33±2.07 days), and unmodified imDC treated group (32.50±5.24 days). (TIF) [file pone.0069640.s008.tif]

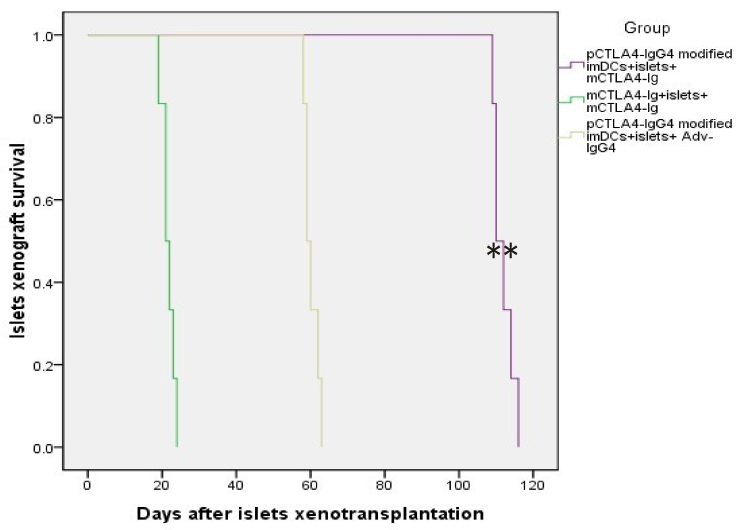

Supplement: Figure S9 — The islet xenograft survival of three groups (Group V, Group VI, Group VII) (n = 6) in the second experiment. Xenograft survival in the pCTLA4-IgG4 modified imDC combined with mCTLA4-Ig treated group (111.83±2.71 days, **P<0.01) was significantly longer than that in the mCTLA4-Ig combined with mCTLA4-Ig treated group (21.67±1.75 days), and the pCTLA4-IgG4 modified imDC combined with Adv-IgG4 treated group (60.17±1.94 days). (TIF) [file pone.0069640.s009.tif]
